# Women at work: Changes in sexual harassment between September 2016 and September 2018

**DOI:** 10.1371/journal.pone.0218313

**Published:** 2019-07-17

**Authors:** Ksenia Keplinger, Stefanie K. Johnson, Jessica F. Kirk, Liza Y. Barnes

**Affiliations:** Leeds School of Business, University of Colorado, Boulder, Colorado, United States of America; Middlesex University, UNITED KINGDOM

## Abstract

Over the last two years, awareness about the sexual mistreatment of women has stunned the world. According to analysis by the New York Times, the defeat of Hilary Clinton and election of Donald Trump spurred a women’s movement in the US that began in November of 2016 and resulted in protests across the country, including the largest single-day protest in history on January 21, 2017. Later that year, the #MeToo movement (starting in October 2017) and subsequent #TimesUp movement (starting in January 2018) galvanized women to unite against sexual assault and sexual harassment, which has become the hallmark of the current women’s movement. But has anything changed over this time period in regard to the sexual harassment of women? Using a repeat cross-sectional survey from over 500 women collected at two points in time (September 2016 and September 2018), we found reduced levels of the most egregious forms of sexual harassment (unwanted sexual attention and sexual coercion) but increased levels of gender harassment in 2018. More importantly, sexual harassment had a weaker relationship with women’s negative self-views (lower self-esteem, higher self-doubt) in 2018 compared to 2016. Qualitative interviews collected from women in the fall of 2016 and in the fall of 2018 from the same women, support the quantitative data. They suggest that the changes in sexual harassment are due to the increased scrutiny on the topic. The interviewees also emphasize that they feel better supported and empowered and are not ashamed to speak up about sexual harassment.

## Introduction

Over the last two years, awareness about the sexual mistreatment of women has stunned the world. According to analysis by the New York Times [1], the defeat of Hilary Clinton and election of Donald Trump spurred a women’s movement in the US that began in November of 2016 and resulted in protests across the country, including the largest single-day protest in history on January 21, 2017. Later that year, the #MeToo movement (starting in October 2017) and subsequent #TimesUp movement (starting in January 2018) galvanized women to unite against sexual assault and sexual harassment, which has become the hallmark of the current women’s movement [2]. The #MeToo movement provided evidence of the pervasiveness of sexual harassment, becoming the largest social movement related to sexual harassment in history with 12 million Facebook posts and 15 million impressions (the number of times the content was displayed) within 48 hours of its inception.

Approximately 80% percent of women in the US report experiencing sexual harassment in the workplace [3], with one study showing that 50% of women had been harassed within the last year alone [4]. The negative effects of sexual harassment are well known and include negative mood, eating disorders, alcohol abuse, job withdrawal, greater stress, greater self-doubt, lower self-esteem, and lower overall mental health [5, 6, 7]. Moreover, the effects of harassment can be seen for nearly a decade following a harassing event [6]. Sexual harassment has largely been conceptualized as a women’s issue; that is not to say that men are not harassed, but we know that men are harassed at a lower frequency and experience fewer negative psychological outcomes of sexual harassment compared to women [5, 8].

The EEOC defines sexual harassment as “unwelcome sexual advances, requests for sexual favors, and […] creating a hostile offensive working environment.” [9]. This definition creates three main categories of sexual harassment: sexual coercion, unwanted sexual attention, and gender harassment [10]. Sexual coercion involves threats toward women who will not comply with sexual requests or bribes in exchange for sex. Unwanted sexual attention reflects sexual advances, including inappropriate comments, staring, and even touching. Gender harassment relates to negative views of women and general gender hostility and, as such, really represents the basis for more egregious forms of sexual harassment and reflects broader gender inequalities in society. Although often seen as the least egregious form of harassment, gender harassment is also the most pervasive and, therefore, can have particularly profound negative effects on women [11].

Although many people experience sexual harassment in the workplace, many never report it [12]. Explanations for the lack or reporting relate to threats to self-esteem and risk of secondary victimization—women fear facing doubts, scrutiny, and blame for the harassment they experience [12, 13]. These fears are captured by stigma theory, which suggests that individuals will avoid sharing a stigma because of self-blame, shame, and fear of negative judgments from others [14, 15]. Stigma theory say that in order to deal with shame, people need sympathetic others who share the same social stigma to feel ‘human’ and ‘essentially’ normal in spite of appearances and in spite of his [or her] own self-doubt” [14] p. 31. Indeed, research on sexual assault shows that disclosing a societally stigmatized experience can affect self-esteem such that positive, validating responses are associated with higher self-esteem whereas negative, blaming, and doubting responses are associated with lower self-esteem [16].

The increase in reporting and public scrutiny of sexual harassment since 2016 could have changed the prevalence of sexual harassment in two very different ways. First, it is possible that increased fear of being punished or having one’s reputation diminished could result in a decrease in sexual harassment [17]. Second, it is possible that increased focus on the topic could lead to a backlash effect that increases sexual harassment as societal members try to maintain existing hierarchies of power [18]. In addition, sexual harassment might have increased because unethical behavior can be contagious [19]. Thus, the exposure to stories of sexual harassment might have influenced the behavior of some individuals in a negative way: “Others also do it, why can’t I?” The #MeToo movement might not only impact those who engage in harassment—it could have also changed the effect of harassment on women. We expect that the increased disclosure and knowledge of the pervasiveness of sexual harassment will buffer women from the negative impact of sexual-harassment on self-views, by demonstrating to women who have been harassed that they are not alone and are supported by other women [20].

We explore these questions using a naturalistic quasi-field experiment of sexual harassment in 2016 and 2018. Using a repeat cross-sectional survey, we compare data on sexual harassment collected from a panel in September 2016 –before the Trump election and #MeToo movement—to data collected from a second panel in September 2018 and examine changes in sexual harassment. We aim to achieve two main goals with these data. First, we test whether there are mean differences in the frequency of sexual harassment over the last two years. Second, we test the relationship between sexual harassment and self-views (self-esteem and self-doubts) over the same time period. In addition to the quantitative data, we conducted qualitative interviews of women in the fall of 2016 and collected data from the same women again in the fall of 2018 to gain insights into why the experience and impact of sexual harassment might have changed over the last two years.

In the quantitative analyses of data collected from over 500 women, we found significantly lower levels of sexual coercion and unwanted sexual attention but higher levels of gender harassment in 2018 compared to 2016. In addition, the negative relationship between unwanted sexual attention and self-esteem was diminished, as was the positive relationship between both gender harassment and unwanted sexual attention with self-doubts. There was no change in the relationship between self-views and the most egregious form of sexual harassment, sexual coercion. Qualitative interviews suggest that sharing one’s story, while knowing that others had experienced the same things, helped the interviewees to feel less ashamed and created increased support and empowerment among women.

## Methods

Study 1: Sexual harassment survey 2016 and 2018. To ensure a 99% chance of finding an effect for the expected interactions, assuming a medium effect size (Expβ1 = 1.3), and .05 R^2^ from other predictors, a power analysis suggested we would need approximately 380 participants. Our final sample comprised 513 women. In September 2016, the authors conducted an online survey using a Qualtrics panel of 250 professional women to gauge the prevalence and impact of sexual harassment. The participants ranged in age from 25 to 45, were working as full-time employees in the US, had an average of 10.6 years of work experience, and the majority were mid-level employees (62%) and White (75%). Participants were paid to participate in a Qualtrics Panel study and were asked questions about their experiences as professionals. In September 2018, we conducted a second survey using another Qualtrics panel. The sample included 263 women who ranged in age from 25 to 45, were working as full-time employees in the US, had an average of 11.5 years of work experience, and were majority mid-level employees (60%) and White (70%). Table 1 provides a summary of the two samples. We controlled for work experience, position level, and race (White/non-White) in the analyses to account for differences. The use of online panels such as Qualtrics is becoming more common in the social sciences and recent comparisons with conventional datasets support the use of the samples [21].

**Table 1:** Comparison of Qualtrics panel samples from September 2016 and September 2018.

	September 2016	September 2018
**Sample size**	250 Women	263 Women
**Age**	25 to 45	25 to 45
**Work Status**	Working full time	Working full time
**Work Experience**	M = 10.6 (SD = 6.1)	M = 10.6 (SD = 6.1)
**Position Level**	22.4% Entry Level 61.6% Middle Level 13.2% Top Level 2.8% Business Owner	23.6% Entry Level 60.1% Middle Level 10.6% Top Level 5.7% Business Owner
**Race**	75% White	70% White

In both waves of data collection, participants were asked to indicate whether they had experienced three types of sexual harassment (gender harassment, unwanted sexual attention, and sexual coercion). Then, they reported on their self-esteem and self-doubts. Both waves of data collection have been approved by the corresponding institutional review board.

### Sexual harassment

Sexual harassment was measured with an eighteen-item measure scored on a five-point Likert scale (0 = not at all, 4 = completely), and measured three types of sexual harassment. Items were summed for each sub-scale [10]. The gender harassment (GH) scale measures general harassment of women which may not be of a sexual nature; for example, the scale asks whether a coworker or supervisor has “made sexist remarks,” and “displayed sexist material.” The internal consistency of the scale was high (Cronbach’s alpha = .90). The second scale, unwanted sexual attention (USA), measures sexual harassment that is more overt—such as “was staring, leering, ogling at you,” and “attempted to stroke/fondle you.” This scale also had a high level of internal consistency Cronbach’s alpha = .95). The final scale assesses sexual coercion (SC) including, “Subtly bribed you with rewards for sexual cooperation,” and “Actually punished you with negative consequences for refusing sexual cooperation” (Cronbach’s alpha = .97).

### Self-esteem

Self-esteem was measured using the Rosenberg Self-Esteem Scale [22], a ten-item measure scored on a five-point Likert scale (1 = strongly disagree, 5 = strongly agree). A sample item is, ‘I feel that I have a number of good qualities.’ The Cronbach’s alpha was .88.

### Self-doubt

Self-doubt was measured with a three-item scale, scored on a five-point Likert scale (1 = strongly disagree, 5 = strongly agree). The scale was developed for this study and assesses self-doubts resulting from the effects of women’s physical appearance on their workplace success. The items were, ‘I wonder whether my success has come from my physical appearance,’ ‘If I were less attractive, I wouldn’t be as successful,’ and ‘My physical appearance makes me doubt my abilities.’ The Cronbach’s alpha was .87.

### Controls

In all of the analyses, we controlled for the position level (from entry level to business owner), years of work experience, and race (White/non-White). Since the self-doubt scale was developed for doubts related to attractiveness, we also controlled for one’s self perceptions of attractiveness (I think I am attractive) in the relevant analyses.

Study 2: Qualitative Analysis. In Study 2, we used a grounded theory approach [23, 24] to collect and analyze qualitative data. Just as with the survey, we collected data at two points in time (in 2016 and 2018). Data collection was approved by the corresponding institutional review board. Initially, we were interested in the downsides of attractiveness for women at work. However, it quickly became clear that we needed to refine our focus and investigate sexual harassment in the workplace as this issue was critical to our participants. We interviewed 31 professional women in 2016. They were chosen through a combination of snowball and convenience techniques [25]. We purposefully targeted women from different industries who have experienced gender harassment, unwanted sexual attention, and/or sexual coercion in the workplace. The first participants were recruited through personal networks and then asked for suggestions about further potential interviewees. Each interview was conducted one-on-one, took between 30 and 90 minutes, was digitally recorded, and transcribed verbatim. At the beginning of each interview, we informed all participants about the goal of the study and obtained verbal consent to take part in an interview.

The participants worked in both female- and male-dominated industries (e.g., social work, corporate law, higher education) and held a variety of roles (e.g., IT specialist, teacher, sales manager). Their age ranged from 25 to 64 (M = 38.4), and 6.5% of them held entry-level positions. Most participants (48.4%) were employed in mid-level positions followed by participants in top-level positions and/or business owners (45.1%). All participants were White. All participants were asked about the downsides of being an attractive woman at work and the relationship between attractiveness and self-esteem. After each interview we wrote memos to reflect on what we were learning from each participant and to document the coding process, code choices, and the emergent patterns in the data [26]. We approached the same 31 women for the second round of data collection starting in September 2018. From the original sample of 31 professional women, we collected responses from 21 women. In the second round of interviews, we focused on sexual harassment and asked whether the women felt that sexual harassment had changed in the workplace over the last two years (and if so—why?) and whether women’s reactions to sexual harassment have changed (and if so—why?). For all of the women who agreed to participate in the second round of reviews, we pulled quotes from their first interview, such as, “In our last interview you talked about being solicited by men and suggested that you thought it was because you smiled a lot.” For quotes like that, we asked them if their impressions have changed over the last two years. Each interview was conducted either in person or over the phone, took between 15 and 30 minutes, was digitally recorded, and transcribed verbatim. At the beginning of each interview, we again obtained verbal consent to take part in the study.

## Results

Descriptive statistics show that gender harassment is the most common type of sexual harassment followed by unwanted sexual attention and sexual coercion. Although the estimates of sexual harassment range from study to study, 87% of women in our sample reported that they had experienced at least one form of sexual harassment. This is similar to other estimates of about 80% [3]. Relatively few women reported that they had experienced sexual coercion, the most egregious type of sexual harassment. In fact, in 2016, 25% of women reported being sexually coerced and in 2018, that number declined to 16% of women. Means, standard deviations and intercorrelations appear in Table 2.

**Table 2:** Descriptive statistics and correlations for 513 female participants collected using a Qualtrics panel (online survey tool). ^1^Race is coded as 0 = not White, 1 = White, ^2^Year is coded as 0 = September 2016, 1 = September 2018. (*P < .05, **P < .01).

	M	SD	1	2	3	4	5	6	7	8	9
1. Year^2^	0.51	0.50	––								
2. GH	5.82	4.76	.05	––							
3. USA	5.85	7.17	-.09*	.82**	––						
4. SC	1.22	3.22	-.18**	.61**	.78**	––					
5. Self-Esteem	3.78	0.73	.16**	-.14**	-.19**	-.25**	––				
6. Self-Doubt	1.96	1.01	-.16**	.43**	.49**	.58**	-.31**	––			
7. Attractiveness	3.51	0.84	-.06	.22**	.23**	.24**	.24**	.30**	––		
8. Work Experience	11.08	6.49	.07	-.06	-.05	-.09*	.10*	-.12**	-.08	––	
9. Position Level	1.97	0.72	.01	.23**	.22**	.26**	.05	.22**	.13**	.14**	––
10. Race^1^	0.72	0.45	-.05	-.01	.00	-.06	-.10*	-.01	-.16**	.10*	.00

**Fig 1:**
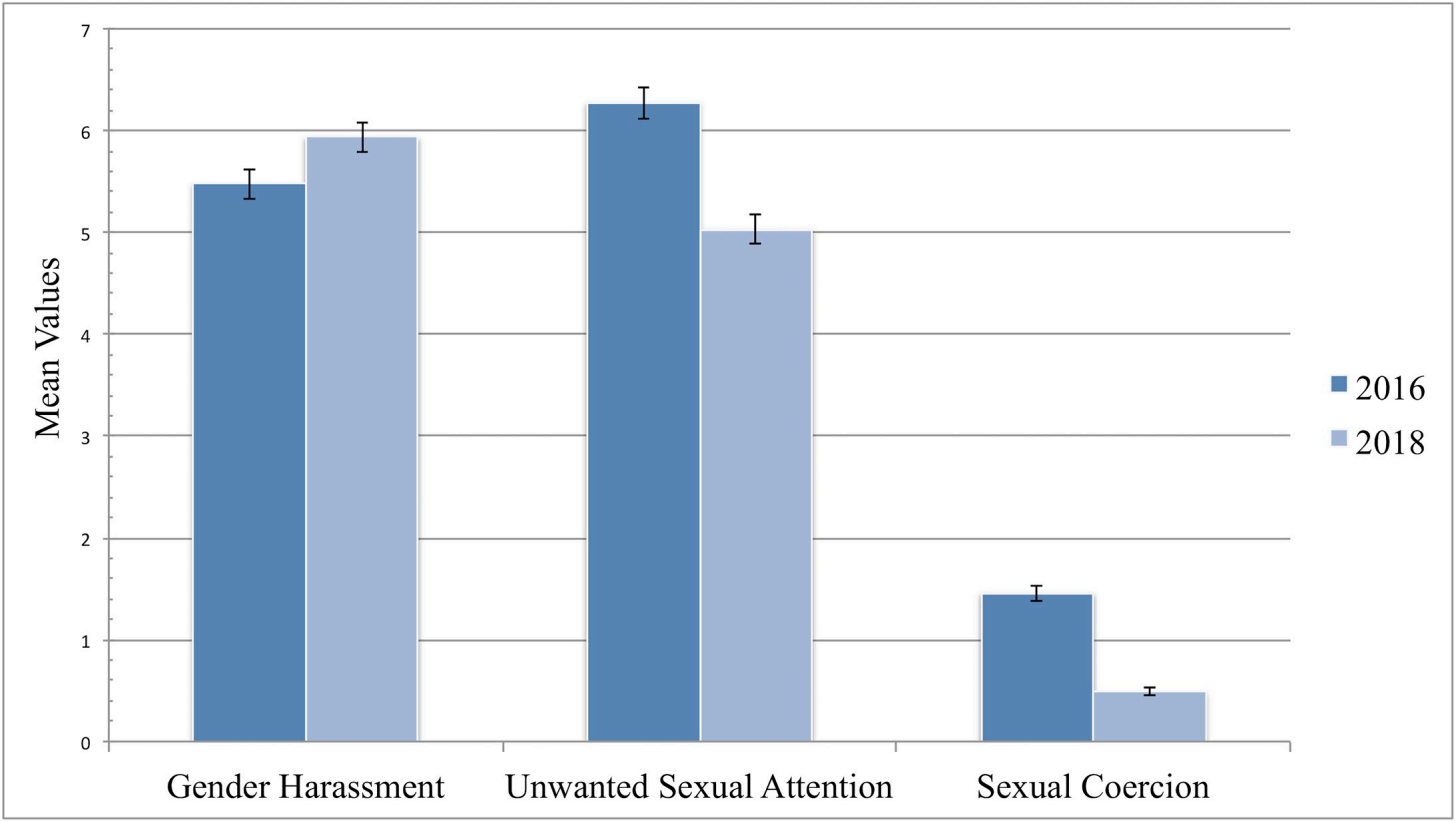
Coefficient estimates for the relationship between year and sexual harassment (sexual coercion, unwanted sexual attention, gender harassment) in 2016 and 2018. Estimates are derived from a Generalized Linear Model using a Poisson distribution of 507 women, 250 of whom were surveys in September 2016 and the remaining women were surveyed in September 2018. We controlled for race (White/non-White), position level, and work experience. Sexual coercion and unwanted sexual attention significantly decrease whereas gender harassment significantly increased over this year.

Quantitative data on sexual harassment from 2016 and 2018. Many of the women in our sample reported experiencing no sexual harassment and, consistent with past research using this scale of sexual harassment [27], the distribution of our data more closely approximated a Poisson distribution than a normal distribution. Analysis with a Poisson distribution is appropriate for data in which there is a large portion of zeros, only on positive tail in the distribution, and where the standard deviation is greater than the mean [28]. To test the changes in mean levels of sexual harassment from 2016 to 2018, we ran a Generalized Linear Model using a Poisson distribution for each of the three types of sexual harassment with the independent variable of year (2016 or 2018). We controlled for the level of one’s position at work, years of experience, and race (White/non-White) and the results are presented in Table 3 and Fig 1. There was a significant and negative relationship between year and both sexual coercion (B = -1.10, P < .001) and unwanted sexual attention (B = -.22, P < .001). In contrast, there was a significant and positive relationship between year and gender harassment (B = .08, P < .05). Although the more egregious forms of sexual harassment appear to be less frequent in 2018, the increase in gender harassment indicates a corresponding backlash effect against women in 2018. It should also be noted that both work experience and position level have a significant effect on all three forms of sexual harassment. While work experience has a negative effect, position level has a positive effect. Race (White/non-White) has a significant negative effect on sexual coercion, suggesting that the non-white participants experience greater levels of this form of harassment. The implications of these effect are explored in the discussion.

**Table 3:** Generalized Linear model using a Poisson distribution to examine changes in sexual harassment across year. The distribution of the sexual harassment variables most closely approximated a Poisson distribution given that the mode was zero, there was a strong positive skew, the data were not bimodal, and the mean and standard error were almost equal. Data are comprised for 513 women. ^1^Race is coded as 0 = not White, 1 = White, ^2^ Year is coded as 0 = September 2016, 1 = September 2018. (*P < .05, **P < .01).

	B	SE	Wald Chi-square
DV = Gender Harassment			
Work Experience	-0.01	0.00	17.22**
Position Level	0.25	0.02	114.28**
Race^1^	0.00	0.04	0.009
Year^2^	0.08	0.04	4.76*
DV = Unwanted Sexual Attention			
Work Experience	-0.01	0.00	22.10**
Position Level	0.37	0.02	247.75**
Race^1^	0.01	0.04	0.08
Year^2^	-0.22	0.04	36.20**
DV = Sexual Coercion			
Work Experience	-0.05	0.01	39.74**
Position Level	0.85	0.05	337.19**
Race^1^	-0.28	0.08	10.46**
Year^2^	-1.10	0.09	147.64**

**Fig 2:**
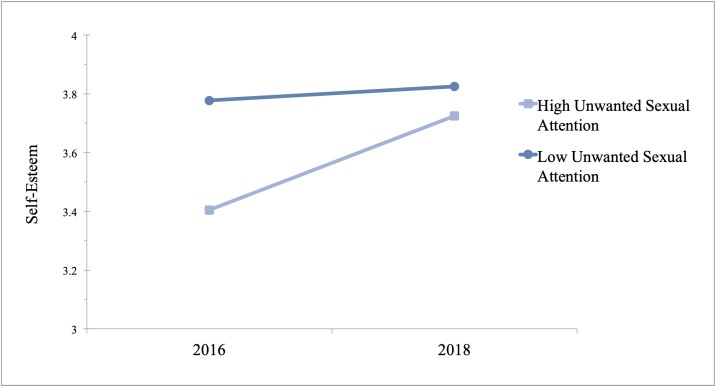
Coefficient estimates for relationship between unwanted sexual attention (± 1 SD) and self-esteem before and in 2018. Estimates are derived from GLM using data from 513 women, 250 of whom were surveyed in September 2016 and the remaining women were surveyed in September 2018.

Next, we examined the relationship between sexual harassment with self-esteem and self-doubts in 2016 and 2018. Because the dependent variable in these analyses were normally distributed, we used the General Linear Model where self-esteem and self-doubt were regressed on each dimension of sexual harassment, year, and their interaction. Again, we controlled for level of one’s position at work, years of experience, and race (White/non-White). To test the nature of the interaction, we used Model 1 of the PROCESS macro designed for SPSS [29]. For self-esteem, there was an overall increase in women’s self-esteem in 2018 (Table 4 and Fig 2). In addition, there was a significant interaction between unwanted sexual attention and year (B = .02, P = .03, 95% CI [.01, .04]). The conditional effects showed that in 2016 there was a significant and negative relationship between unwanted sexual attention and self-esteem (B = -.03, P < .001, 95% CI[-.04, -.02]), but in 2018 this relationship was non-significant (B = -.01, P = .33, 95% CI [-.02, .01]). There was not a significant interaction between year and sexual coercion nor gender harassment in predicting self-esteem.

**Table 4:** General Linear Model regressing self-esteem and self-doubt on the relationship between year and sexual harassment. Unwanted sexual attention has a less negative relationship with self-esteem in 2018. Gender harassment and unwanted sexual attention were less positively related to self-doubt in 2018. ^1^Race is coded as 0 = not White, 1 = White, ^2^ Year is coded as 0 = September 2016, 1 = September 2018. (*P < .05, **P < .01).

DV = Self-Esteem				DV = Self-Doubt			
	B	SE	T		B	SE	T
Work Experience	.01	.01	1.89	Work Experience	-.02	.01	-2.50*
Position Level	.06	.05	1.43	Position Level	.19	.06	3.40**
Race^1^	-.17	.07	-2.47*	Race^1^	.08	.09	0.94
Gender Harassment	-.03	.01	-3.64**	Attractiveness	.24	.05	4.94**
Year^2^	.12	.10	1.17	Gender Harassment	.09	.01	8.35**
YearXGH	.02	.01	1.30	Year^2^	-.10	.12	0.46
				YearXGH	-.04	.02	-2.20*
Work Experience	.01	.00	1.96*				
Position Level	.06	.05	1.44	Work Experience	-.02	.01	-2.72**
Race^1^	-.18	.07	-2.55*	Position Level	.18	.05	3.33**
USA	-.03	.01	-4.67**	Race^1^	.08	.08	0.91
Year^2^	.07	.08	0.89	Attractiveness	.22	.05	4.74**
YearXUSA	.02	.01	2.17*	USA	.07	.01	9.92**
				Year^2^	-.06	.10	-0.58
Work Experience	.01	.00	1.61	YearXUSA	-.03	.01	-2.38*
Position Level	.11	.05	2.33*				
Race^1^	-.19	.07	-2.76*	Work Experience	-.01	.01	-2.17*
Sexual Coercion	-.06	.01	-4.99**	Position Level	.11	.05	2.02*
Year^2^	.15	.07	2.18*	Race^1^	.12	.08	1.52
YearXSC	-.01	.02	-0.33	Attractiveness	.20	.04	4.52**
				Sexual Coercion	.15	.01	11.39**
				Year^2^	-.11	.08	-1.39
				YearXSC	.02	.03	0.62

**Fig 3:**
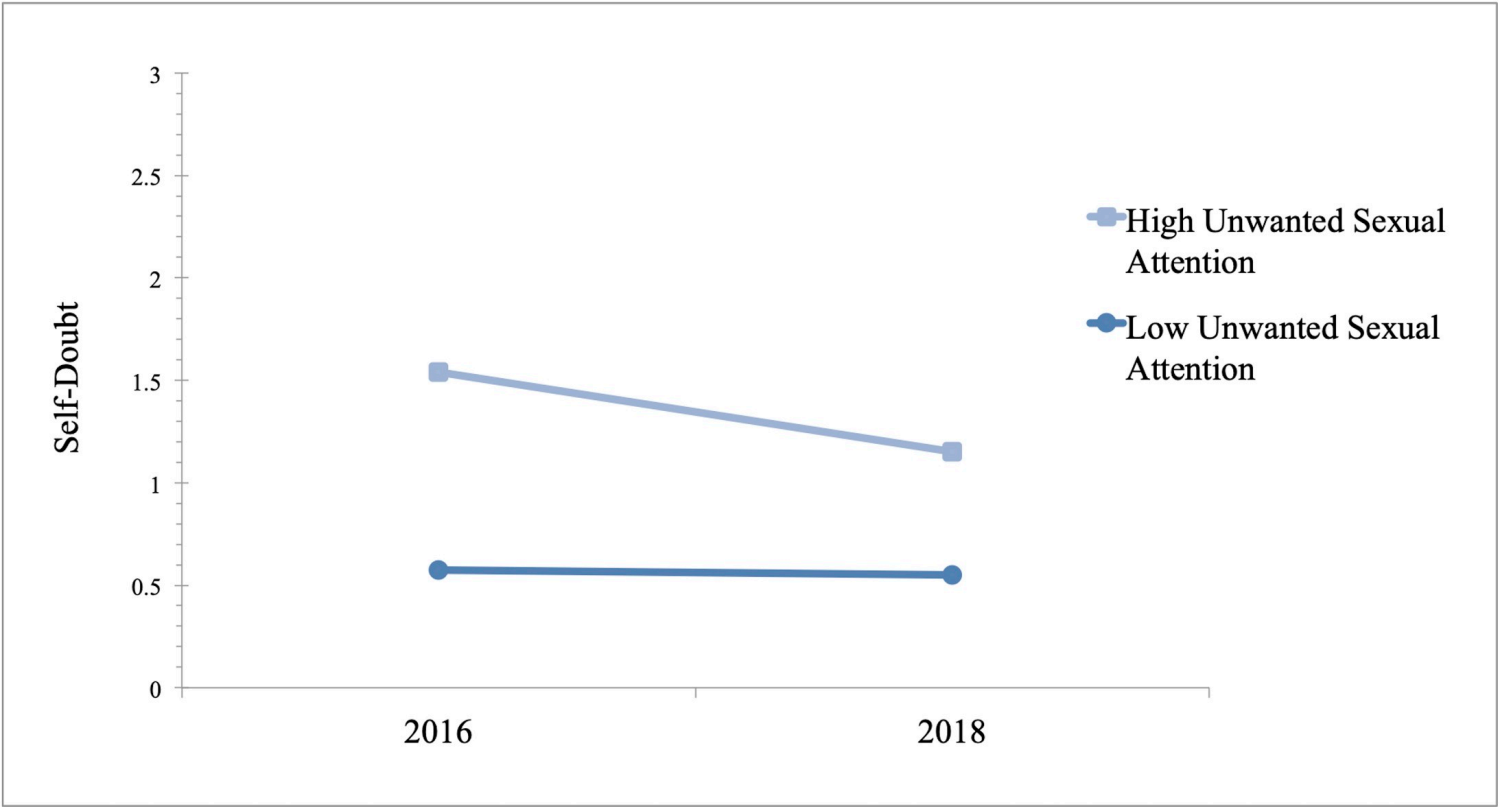
Coefficient estimates for relationship between unwanted sexual attention (± 1 SD) and self-doubt in 2016 and in 2018. Estimates are derived from GLM using data from 513 women, 250 of whom were surveyed in September 2016 and the remaining women were surveyed in September 2018.

**Fig 4:**
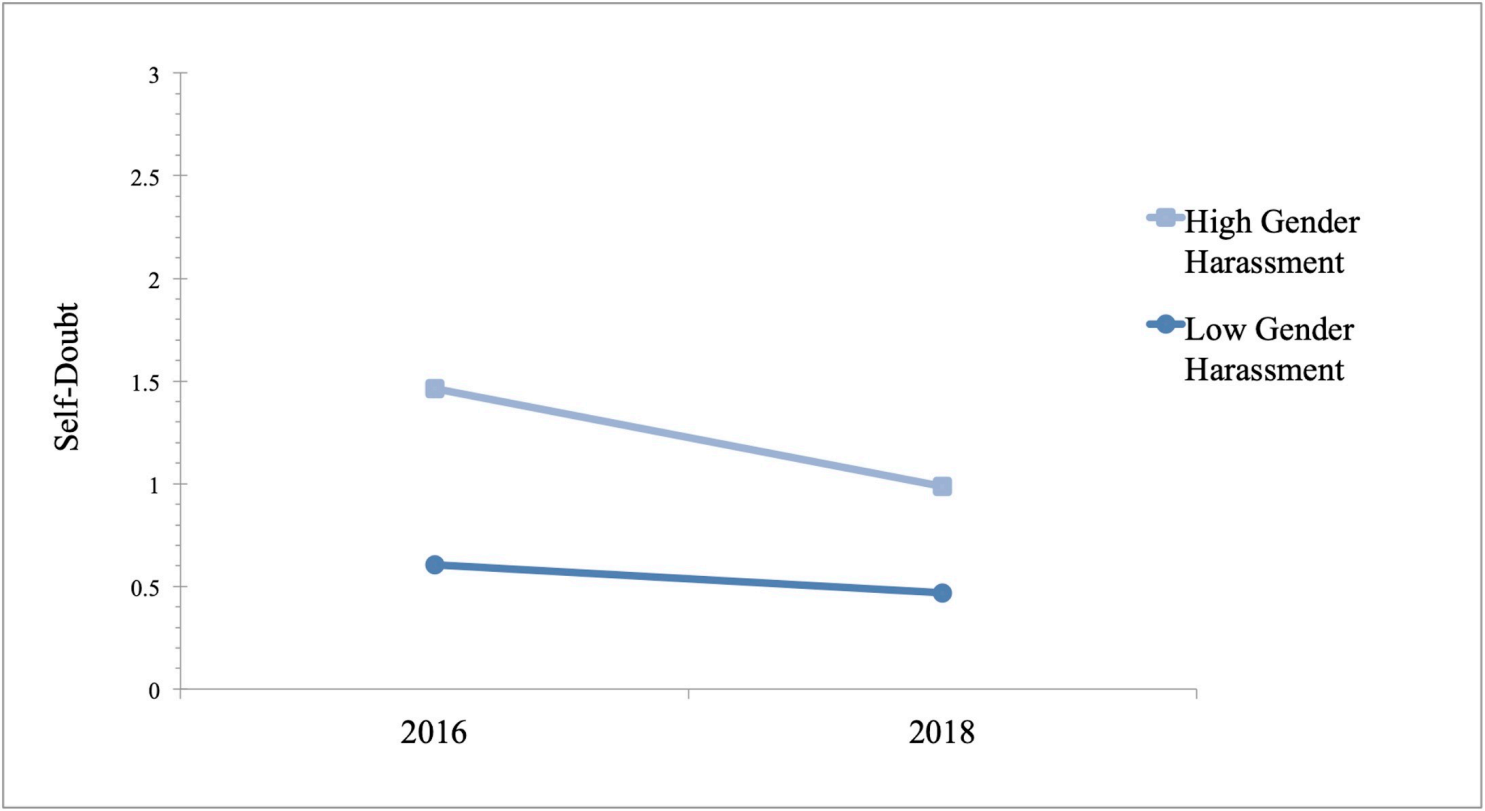
Coefficient estimates for relationship between gender harassment (± 1 SD) and self-doubt in 2016 and in 2018. Estimates are derived from GLM using data from 513 women, 250 of whom were surveyed in September 2016 and the remaining women were surveyed in September 2018.

We conducted the same analyses for self-doubt but added an additional control variable related to one’s perception of their own attractiveness (I am physically attractive) because our self-doubt measure centers around wondering if a woman’s success is attributed to her looks. Without controlling for this variable, the results were not significant. Like self-esteem, there was a significant interaction between unwanted sexual attention and year in predicting self-doubt (B = -.03, P = .02, 95% CI [-.05, -.01]). The conditional effects showed that in 2016, there was a positive relationship between unwanted sexual attention and self-doubt (B = .07, P < .001, 95% CI [.05, .08]) and that this relationship was still significant in 2018, although the effect was smaller (B = .04, P < .001, 95% CI [.03, .06]). In addition, the interaction between gender harassment and year predicted self-doubt (B = -.04, P = .03, 95% CI [-.07, -.01]). The conditional effects showed that gender harassment was positively related to self-doubt in 2016 (B = .09, P < .001, 95% CI [.07, .11]) and in 2018, but the effect was reduced (B = .05, P < .001, 95% CI [.03, .08]). The results are also presented in Table 4 and Figs 3 and 4.

In sum, the results of the quantitative study show decreased reports of unwanted sexual attention and sexual coercion, but increased reports of gender harassment. The negative relationship between unwanted sexual attention and self-esteem and the positive relationship between both unwanted sexual attention and gender harassment and self-doubts were both lower in 2018 than in 2016.

## Sexual harassment interviews 2016 and 2018

We complement our quantitative data with qualitative interviews collected in 2016 and in 2018. We coded and analyzed the data as they were collected using a grounded theory approach [23, 24]. First, two independent researchers performed line-by-line open coding by reading through the data multiple times and generating a large number of conceptual labels in NVivo 11. In addition, we open coded field memos that were written after each interview. Examples of open codes include, “I get a lot of sexual advances that are not necessarily wanted” and “I had a guy ask me if I’ve had any work done up there.” The next step involved axial coding—reassembling the data in ways that allowed us to explore the relationships between and within categories [24]. We identified the most important and frequent concepts and grouped them into more generalizable categories (e.g., sexual coercion, reporting sexual harassment, self-doubts, etc.). Contacting several participants after interviews to clarify the emerged concepts helped us to refine the categories. We kept collecting data and coding them until we reached the point of theoretical saturation at which the new data did not provide any new information [30]. In our case, no new codes emerged from the data after the 30th interview, and data collection ceased after the 31st interview. Because the initial interviews were very broad (the experiences of attractive women at work), themes that were unrelated to sexual harassment also emerged (e.g., others doubting one’s competence). Because we were particularly interested in sexual harassment, we do not include those data here and we focused our second round of interviews (2018) exclusively on sexual harassment and the #MeToo Movement.

The findings from the 2016 interviews suggest that even though most women reported being sexually harassed, only few of them ever reported it out of fear of negative repercussions. Longer quotes are in Appendix A, but one woman said, “I didn’t complain about him. HR departments—I never trust anyway” [a top-level executive, 40+, Public Relations]. Another participant added that she could not complain about someone who has made explicit sexual comments to her because “he was this fairly famous guy in this field. It was very uncomfortable” [a college professor, 40+, higher education]. A second theme that emerged in the 2016 interviews was that women blamed themselves, to at least some extent, for being sexually harassed. For example, after receiving unwanted sexual attention, one woman said, “I started questioning myself, I was like, ‘Did I give him that vibe?’” [a marketing manager, 30+, marketing and advertising]. Another participant admitted that her former boss made her doubt herself by telling her, “Oh, I thought you were so hot. That’s why I hired you” [a lawyer, 30+, tech industry].

We re-interviewed 21 of the same women in 2018 to ask their impressions of what, if anything, has changed over the last two years. With the focus on sexual harassment, most women discussed the changes they had experienced in relation to the #MeToo movement. Two of the themes that emerged related to women’s impressions of if and how organizations and men have changed over the last two years. Women believed that organizations are taking sexual harassment more seriously (e.g., “I think it’s making them think twice when those issues are being brought up and what do they do about it, instead of just sending a guy to some sort of diversity or sensitivity training” [a consultant, 30+, engineering]). In addition, women noticed clear changes regarding men’s behavior: “You aren’t getting told anymore, sleep with me or you’re losing your job” [a top level executive, 40+, tech industry]. Another woman added, “it [sexual coercion] went down just because people are more aware that it’s not okay anymore or women started to talk more and then maybe they [men] will keep their hands to themselves as a result” [a software engineer, 30+, IT]. At the same time, the participants believed that some men became more aggressive and hostile towards women in the workplace after the #MeToo movement: “I think there’s increased hostility towards the women who have been empowered by the #MeToo movement who aren’t as quiet about it anymore and just coming out and saying, ‘That’s not okay’” [a college professor, 30+, higher education]. According to another interviewee, “now it [sexual harassment] is just under the covers a little bit more and it’s just innuendos” [a top level executive, 40+, tech industry]. These findings seem consistent with our quantitative findings of decreased sexual coercion and increased gender harassment after the #MeToo movement.

Finally, there were three themes related to women’s own experiences with sexual harassment after the #MeToo movement. The first theme was related to self-blame and shame. The interviewees emphasized that the #MeToo movement helped many victims of sexual harassment not to feel ashamed anymore: “The Me Too Movement is finally making it so that people are comfortable enough to say it out loud, because they are understanding that it was wrong and that it wasn’t their fault” [a sales manager, 30+, marketing]. The second theme that emerged from our interviews was the support from other women: “If you can do the thing where you actually go to even just one woman and say, ‘This happened to me’ and get encouragement […], then you’ve got a chance” [a top level executive, 40+, tech industry]. Related to this, the third theme was about empowerment after the #MeToo movement: “I have a sense of a little bit more empowerment in women around me, and that’s good” [a college professor, 50+, higher education]. These quotes, in their entirety, and other related quotes appear in Appendix A.

Stigma theory suggests that people experience negative outcomes of stigma because of self-blame, shame, and fear of negative judgment but that sharing with sympathetic others who have had similar experiences can reduce shame [14]. Our results suggest that seeing women share their stories created an opportunity for support in the form of bonding and community with other women. In addition, women emphasized feeling empowered and not ashamed to share their experiences with sexual harassment in the workplace. They also said that organizations were taking the topic of sexual harassment more seriously and that men may have changed their attitudes towards sexual coercion and gender harassment.

## Discussion

The women’s movement that has emerged over the last two years will hold a profound place in history, particularly in relation to the sexual harassment of women. Yet, its impact has not been empirically examined, to date. Although we cannot prove causality, this study offers the first attempt to assess changes in the experience of sexual harassment for women in the workplace over the last two years. In our sample, 87% of women reported experiencing some type of sexual harassment. Women reported lower mean levels of sexual coercion and unwanted sexual attention in 2018, but a higher mean level of gender harassment compared to in 2016. From a theoretical perspective, it is possible that sexual harassment has declined in the workplace because of increased fear of being punished or having one’s reputation diminished [17]. The fact that the most egregious form of harassment has declined appears to be good news on the surface; but gender harassment can have an equally negative impact on women because of its pervasive and continued nature and could reflect backlash against women in an effort to maintain the gender hierarchy in society [11].

More importantly, we examined changes in the relationship between sexual harassment and women’s self-views. Consistent with research showing negative emotional effects of sexual harassment on self-esteem, self-doubt, and other mental health outcomes [5, 6, 7], we found significant relationships between sexual harassment and negative self-views; but these relationships weakened in 2018. Specifically, we found a significant decrease in the negative relationship between unwanted sexual attention and self-esteem. In addition, the positive relationship between unwanted sexual attention and self-doubt declined, just as the positive relationship between gender harassment and self-doubt did. In the past, low levels of reporting sexual harassment have been attributed to women’s fear that they will face doubts, scrutiny, and blame for the harassment they experience [12, 13]. Indeed, stigma theory suggests that we often hide stigma for these reasons [14, 15]. However, the theory also notes that access to sympathetic others who share the same social stigma allow people to feel more normal despite their own self-doubt [15]. There is strong evidence that disclosing an experience that is highly stigmatized by society can improve self-esteem when people feel validated and supported [16]. This could very well be what happened in response to the #MeToo movement. Seeing the accounts of so many women’s sexual harassment experiences could have reduced the social stigma, allowing people to share their own experiences and feel validated when others provided support and understanding.

Interestingly, we did not see a reduced relationship between sexual coercion and self-views. This could be explained by the relatively low frequency of sexual coercion and the highly significant relationship between sexual coercion and self-views. It is possible that the impact of sexual coercion on women’s self-views will take much more time to change. The effects of sexual harassment can be seen nearly a decade following harassment which might suggest that even if the current women’s movement has benefitted women, it may take some time for these effects to emerge [31]. In our qualitative analysis, we looked for reasons why sexual harassment may have changed in the last two years. Women attributed the perceived change in sexually harassing behaviors to the increased visibility of the issue. They also emphasized that the #MeToo movement helped many victims of sexual harassment not to feel ashamed anymore and to switch the focus from the victim to the perpetrator. Knowing that so many other women had been harassed and were brave enough to share their stories reduces the stigma of sexual harassment, consistent with stigma theory. The awareness of people who share the same social stigma and who are willing to share it openly creates feelings of acceptance, support, and validation [14, 15]. Women also reported feeling supported by other women and empowered as a result of the current women’s movement.

Although it was not the primary focus of our study, the significant effects of work experience, position level, and race are interesting and warrant a brief discussion. In the main effect models, work experience had a significant negative effect on all forms of sexual harassment, while position level had a significant positive effect. These results support contrasting theories of power and sexual harassment. On the one hand, lacking institutional power by lacking work experience or being non-White, can make one more vulnerable to sexual harassment when harassers use their positions of power to extort sex from women [32, 33]. As such, being low in work experience and being non-white were both related to greater harassment (race was only significantly related to sexual coercion). In contrast, theories of power threat suggest that as women gain more organizational power (such as being higher in position level) they are more likely to be harassed as a means to denigrate powerful women and maintain the patriarchy [27, 34]. The simultaneous existence of the two mechanisms of sex and power is intriguing and offers an interesting area for future research explaining the potential for non-white women’s intersectionality to make them particularly vulnerable targets of harassment.

There are, of course, limitations to this research. The data are purely correlational and although we collected data before the #MeToo movement and in 2018, we cannot assert that the current women’s movement caused the changes we observed. However, if the changes were due to demand characteristics, the differences we observed were due to perceptions alone, we would likely expect that we would see universal increases or decreases across all three types of sexual harassment. For example, women might expect that sexual harassment should have decreased as a result of the #MeToo movement, so they might report reduced levels of sexual harassment even if no changes occurred. Conversely, the public focus on how many women have been harassed, may cause them to erroneously think there is more sexual harassment just because they are more aware of it. If either explanation were true, we would likely see an overall change in sexual harassment between 2016 and 2018, rather than declines in reports of some types of sexual harassment (sexual coercion and unwanted sexual attention) and increases in gender harassment. As a result, we believe that there have been actual changes in sexual harassment, although our data cannot prove that is the case. Related, a strength of the study is the use of data collection before and in 2018, rather than using retrospective accounts, which are sensitive to a wealth of biases [35, 36].

A second limitation is that we only collected data from women, so we do not have men’s perspectives as potential harassers or harassees. Although we do know that men experience sexual harassment, we also know that they are harassed at a lower frequency and that the experience of harassment does not result in as many negative psychological outcomes [8]. Nonetheless, this is a limitation of our study. Further, although online samples like we used in the quantitative study are increasingly common [21], the panels created by organizations like Qualtrics are not random samples. For example, they might exclude individuals who do not have access to a personal computer to complete an online survey. We also recognize that the ordering of our questions in the quantitative study is a limitation and we should have used counterbalancing to eliminate the possibility of order effects. While we do not believe these concerns represent a threat to the overall findings, it is a limitation that should be addressed in future research.

In sum, the findings from this study provide suggestive evidence that sexual harassment might be changing. The data from the qualitative interviews suggest that women feel greater support from their peers and believe that the increased scrutiny on the topic has decreased the most egregious sexual harassment behaviors. Each of these insights points to the importance of continued attention toward sexual harassment in the workplace and the importance of providing additional support for those who experience harassment.

## Appendix A—Quotations

### 2016 quotes. Theme: Women question themselves

One day [my co-worker] said to me, ‘Do you want to go get a glass of wine together after work?’ I was like, ‘No.’ The next week he just flat out said, ‘You know how I feel about you, and I think you feel the same way.’ It was one of those moments when you remember everything because you are just so hyper aware and shocked. I started questioning myself, I was like, ‘Did I give him that vibe?’ [a marketing manager, 30+, marketing and advertising].

My mom came and met me in Boston for this conference. We went to the event at night, where there’s drinking and stuff. These guys were hitting on me. My mom’s there. I look at them and I go, ‘WTF. Why? What am I doing?’ [a top level executive, 40+, tech industry].

There’s always situations where you kind of feel like you’re being objectified or like you kind of have to question like, “Did I get this for the right reasons?”[a front office assistant, 20+, dental services]

Yeah, I think that if a woman feels that she’s an attractive person and has that reinforced by others, I can see that it would make her feel self-conscious and it would make her “Oh, did they ask me to do this because …” Kind of like my scenario with my coworker where he was very forward with me. I question why he mentored me before this exchange because of … Did he do that because he was trying to get in my good graces for something else to happen? Does he actually find value in me as a team member and as a professional, or were his intentions just that? It definitely brings questions [a teacher, 20+, early childhood education].

I think that a lot of women in our society are going to feel self-conscious or question their own merit […]. Our culture places so much emphasis on the sexualization of women’s bodies [a college professor, 40+, higher education].

[…] it [unwanted sexual attention] also then affects my confidence, because is that why they’re asking me to dinner? Do they actually care about what I have to say? Am I going to sit there and just have to listen to these men? […]. Well, it questions my value. It makes me question why am I really at the table [a college professor, 30+, higher education].

### 2016 quotes. Theme: Women do not report sexual harassment

At lunch, it was the three of us at the table, and [a colleague] asked us, ‘Where are you staying? That’s like a girls’ pajama party, you all staying at the same place. What do you wear to your girls’ pajama party?’ You want to nip it in the bud early so that you don’t lose a customer over it, you don’t lose an opportunity over it, and you don’t get put in a position where you have to really shut it down. With that, I no longer work with that customer anymore, because I just didn't want to have to deal with him. Is that fair? No, but life is too short [a top level executive, 40+, tech industry].

We worked with really beautiful models and we worked with these two twins. The executives were so vulgar about these twins. They wouldn’t leave them alone. The chief legal officer …cornered a twin in the hallway, he was reciting poetry to her. He would not leave her alone. There was a woman at the company who insisted that I report it to HR because she knew it would be the death of my career. I did not report it. HR departments—I never trust anyway. They’re the most gossipy people in any organization. I talked to [the model] about it. She didn't want it to be escalated in any way. She wants to keep her job as a model with the company [a top level executive, 40+, Public Relations].

I’ve definitely been in situations where I’ve felt sexually harassed. A couple of years ago, I was wearing a sweater dress and some tall boots, and I had this one male colleague make some comment about it. I think he said I look like an alluring zorro or something… which really creeped me out. He’s senior to me so I didn't feel like I could say anything. He said in front of another woman who I was talking to, and we both kind of looked at each other and was like, ‘What? Did he really just say that?’ … I should have said something to my boss, but I didn’t want to make things weird. I feel like this guy gets away with things [an urban planner, 30+, local government].

He’s driving me home back into the city and he says under his breath he’s in the car and he’s like kind of grinding his choppers and he’s like, ‘I'll bet you’re really nasty in the bedroom,’ and I was like, ‘Did I just hear that right?’ He’s like, ‘Whips and chains and high heeled boots,’ and I was like, ‘Did I just hear that correctly?" I’m in this car with him going through Years Square and I want to dive roll out of the car but I’m just going to pretend I did not hear that. I just totally dismissed it and changed the subject. Then I went back to the office and was saying to [a female colleague], ‘Oh my god [person’s name] just said this stuff.’ She was like, ‘Yeah.’ He was a client. I can’t report it to my HR. I don’t have anyone to really go to [a top level executive, 40+, Public Relations].

I’ve had bosses come onto me, and you don’t want to get fired. […] when bosses hit on me or flirt with me I just ignore it [a lawyer, 60+, defense law].

I would say, my boss was flirty, like an outwardly flirty. Sometimes things would be inappropriate. I would call him on it. I would be like, ‘Really, did you just say that?!’ But he took it, he thought I was like joking. […]. I just would blow it off [a PR manager, 30+, Public Relations]

### 2018 quotes. Theme: Organizations take sexual harassment more seriously

I think the Me Too movement made folks at the administrative level say, ‘We have to be doing this.’ At the most basic level we need to at least be taking our sexual harassment training [a college professor, 40+, higher education].

I just feel like it is more common, it’s more accepted, there are clearer paths with which to take those sorts of complaints. I know what options to go to at the university or who to talk to and HR and whatever supposedly provides an outlet for to share the thing. So I think that definitely universities and I know a lot of other corporation type entities are spelling out really clear pathways for people to do it [an educator, 30+, higher education].

I think there is broader awareness of the fact that sexual harassment etc. in the workplace is a thing, so I think more broadly, culturally it seems like more people are aware [an educator, 30+, higher education].

I would say it’s [sexual harassment] more of a topic of conversation in the workplace now than it was before. I do hear more conversation around what is and is not appropriate whereas before that conversation seemed to be more behind closed doors about correcting behavior when it got out of line rather than being proactive and talking about what was appropriate to begin with [a defense contractor, 30+, arms industry].

I think it’s making them think twice when those issues are being brought up and what do they do about it, instead of just sending a guy to some sort of diversity or sensitivity training. Maybe we actually need to take a bolder step here [a consultant, 30+, engineering].

I think people became more aware that it's a widespread thing. I mean women always knew it's a widespread thing but I think men became more aware that it's happening around them. That men who haven't harassed anybody but didn't know that others were harassed [a software engineer, 30+. IT].

### 2018 quotes. Theme: Changes in sexual coercion and gender harassment

There is fear and the fear has paralyzed people on some levels […]. The fear part is subsiding and now it’s turning to anger. It was rather disturbing […]. At least in the workplace there’s definitely this sort of anger towards women that’s cropping up [a lawyer, 30+, tech industry].

Some of the people who don't see it as an issue are doubling down and getting more aggressive. They feel like it’s really just a politically correct movement that’s taken over their freedom to speak and just be people in the workplace […]. I think there’s increased hostility towards the women who have been empowered by the Me Too movement who aren’t as quiet about it anymore and just coming out and saying, ‘That’s not okay.’ I think there’s some hostility towards those women in particular [a college professor, 30+, higher education].

I don’t know that the Me Too movement has really had impact on the everyday microaggressions … We just had our bystander training and [a man] went up to his project manager…and he says to her, ‘I found your name tag on the floor.’ She says, ‘Really?’ He goes, ‘Yes,’ and handed her a sugar packet. I felt like most of what was publicized were big things, not the little things on a daily basis that really undermine the confidence and the belongingness for a women in engineering [a college professor, 40+, higher education].

I would hope that it [sexual coercion] went down just because people are more aware that it’s not okay anymore or women started to talk more and then maybe they [men] will keep their hands to themselves as a result. That’s the hope anyway [a software engineer, 30+, IT].

You aren’t getting told anymore, sleep with me or you’re losing your job. But now it’s just under the covers a little bit more and it’s just innuendos…This guy [asked a friend], ‘How did you get mentored by this author?’ She said, ‘It’s called networking.’ And he said, ‘Don’t ever talk to me that way again or I will smack you in the face.’[a top level executive, 40+, tech industry].

I think that there’s also still a really significant component in our culture that hasn’t shifted and maybe has become more entrenched, more dug in, about relationships between men and women and in the workplace as a result [a college professor, 40+, higher education]. I feel like we’re under such attack…I'm optimistic for women that they can speak out now more than ever before. I’m optimistic that maybe some of these guys that were sexually harassers will stop. I think actually as long as Trump is in power, they’re still emboldened to be more even more aggressive…There’s this Trumpism that is like, ‘we gotta hold on because the White man is under attack.’[a top level executive, 40+, Public Relations].

### 2018 quotes. Theme: Feeling shame and self-blame

The Me Too Movement is finally making it so that people are comfortable enough to say it out loud, because they’re understanding that it was wrong and that it wasn’t their fault and they’re finally comfortable enough to say it out loud. [a sales manager, 30+, marketing].

I think it [sexual harassment] just became from a taboo subject, now it’s more in the open. I was maybe raised in a culture where the explanation like, ‘Oh your skirt is too short, that’s why’ has definitely been a prevalent argument brought up. Like you brought it on yourself. So I think that that’s where my mind would go. Now I’m pissed. [a software engineer, 30+, IT].

I’m on this Facebook group. It’s female lawyers; there’s probably a couple hundred women on it. There’s all these posts about being sexually assaulted. I think that is a good benefit of it because it takes the shame off of the victim and puts it onto the perpetrator. It used to be like, ‘Well, why were you out at two in the morning? You must have just been doing something wrong.’ Now I think the public is shifting towards putting the attention where it should be, which is disgust and anger towards the perpetrator. [a college professor, 30+, higher education].

I was like, ‘I just need to tell people this.’ Instead of worrying who my Facebook friends are, instead of worrying what are they going to think, I was like, ‘how did this affect me? Who cares how this could affect them? This was something that happened to me.’ [a marketing manager, 30+, marketing and advertising].

It definitely feels like people are more willing to talk about it and realizing that, they’re not to blame. It’s someone else’s fault. [a sales manager, 30+, marketing].

What I do think is I think it may bring an awareness, meaning the awareness to say, ‘Okay, yeah. This did happen to me and maybe it is time to seek somebody out to talk to about it.’ [a college professor, 40+, higher education].

I think there is this like bonding for me to be able to tell that story and not feel ashamed because, you know, in my head, I’m like, ‘She’s probably thought I was flirting.’ It just has changed everyone’s perceptions, I feel like. [a marketing manager, 30+, marketing and advertising].

I would say there’s less shame. I had this really bad experience when I was at home a couple weeks ago. My stepfather and my brother were defending Kavanaugh and they were saying, ‘If Dr. Blassey Ford, if this happened to her, why didn’t she tell her parents? Why didn’t she tell anyone?’ And I said, ‘I was sexually assaulted in college and I didn’t tell anybody, do you wanna know my story? And I never told you, so I will tell you now.’ I felt like emboldened to be able to tell the story because I’m not alone and like there’s women all over the place telling their story. [a top level executive, 40+, Public Relations].

### 2018 quotes. Theme: Getting support

I actually didn’t want to do it at first. But I started seeing them [#MeToo posts] coming in and I was just like ‘Oh my gosh they’re being so brave. Telling very personal stories that I never knew about.’ I’ve been so proud of all of my friends, my family friends that are women, family that are women, husbands, etc. that I wanted to do it, too. And I never would’ve done it if I hadn’t felt that safety to do it. […]. I think it’s that women are empowered by bonding and community. We’re very social creatures and you don’t feel like you’re alone. Women are standing up for you. [a marketing manager, 30+, marketing and advertising].

Community is such a big deal and to talk about it. If you can do the thing where you actually go to even just one woman and say this happened to me and get encouragement, then it kind of feeds on itself, then I think, if you don't stay isolated, then you’ve got a chance. [a top level executive, 40+, tech industry].

That’s why community is such a big deal and to talk about it. If you can do the thing where you actually go to even just one woman and say this happened to me and get encouragement. Then it kind of feeds on itself if you don't stay isolated. It isn't like I’m vindicated, it is more, I’m validated. [a top level executive, 40+, tech industry].

It’s subtle, it’s very, very subtle, but I think that women are bonding more. I saw that with my friend, that she and I will talk a lot about it. And just bonding over that, like, ‘Wow, I’m gonna say something. That’s not appropriate.’ I’m feeling empowered. [a marketing manager, 30+, marketing and advertising].

It was a discussion around action and it was this discussion around what we could be doing differently, you know, as we walk into these big meetings and sitting at the table and taking a chair that was closer. It’s just so much of it. I felt like that created almost a greater support network and it was this support network [a college professor, 40+, higher education].

I gave this advice at the class I taught last week. They were like, ‘Do you have any advice for women who are experiencing harassment in the work place?’ I said, ‘Yes, safety in numbers. Find other women who have been harassed by that person and work together to bring it up to a higher power together.’ [a top level executive, 40+, Public Relations].

### 2018 quotes. Theme: Feeling empowered

I have a sense of a little bit more empowerment in women around me, and that’s good, and more conversations that are good. [a college professor, 50+, higher education].

I think women seem to be talking up more about it when things come up in a meeting or when things come up in conversation. We feel a little bit more empowered to say what we think and what we feel about harassment and discrimination, as opposed to before when you were like, ‘Well, I don't know if I should talk about this.’ [a college professor, 50+, higher education].

I have remembrances of men saying obnoxious things in a meeting to a female colleague of mine and nobody calling them out on it, whereas now…if it happens and I’m in a meeting, I will certainly call it out now, for sure. [a college professor, 50+, higher education]

I think it [MeToo movement] has brought to light the fact that this isn’t a one-off thing, and by the way, a lot of women are impacted by it, which I think helps to get … help women who have been impacted by some sort of event that's happened in their life to speak out about it, and bring it forward. So, I think that it helps with empowerment [a consultant, 30+, engineering].

They responded with, ‘We’re gonna make sure other people were able to say this happened to you and I’m gonna make sure you know I’m in your camp. I’m an advocate for you, I support you.’ She psychologically says if I speak up, I have power now. If they treated her nice like that, she’s walking around going what I say matters. I can speak up and stand up for myself. I can advocate for myself. I didn’t get that [when I was harassed] [a top level executive, 40+, tech industry].

Just being able to put a name to it and not feeling like ‘Oh my gosh, am I overreacting?’ And realizing it, and seeing that it’s making a difference. It just makes you feel like you are empowered, I think. I think that it was an empowerment movement because I think that maybe a few years ago and in my 20s, I would’ve said ‘Oh, I was being so silly, I misunderstood.’ Internalizing it, maybe telling the story to my friends. They would agree [a marketing manager, 30+, marketing and advertising].

I do have hope for some of the millennial ladies …I was just traveling to DC on business a week ago with two millennials and one of them is 25, and she told me she and her roommates have a women empowerment room in their house. It’s got pictures of all women that they admire. Michelle Obama and Hilary Clinton and Amelia Erhardt and Ruth Ginsberg and Anita Hill, all in this empowerment room and then they have these comfy chairs and it’s their zen room and they go there to restore [a top level executive, 40+, Public Relations].

## Supporting information

S1 File Interview protocol for 2016. Interview questions and protocol for 2016 qualitative interviews.
(DOCX)

S2 File Interview protocol for 2018. Interview questions and protocol for 2018 qualitative interviews.
(DOCX)
