# Supplementary material for: Women at work: Changes in sexual harassment between September 2016 and September 2018
Source: PLoS One. 2019 Jul 17;14(7):e0218313. doi: 10.1371/journal.pone.0218313 (PMC6636712; doi:10.1371/journal.pone.0218313)
Supplement: S1 File — Interview questions and protocol for 2016 qualitative interviews. (DOCX) [file pone.0218313.s001.docx]

University of Colorado at Boulder

LEEDS School of Business

**Interview Protocol**

| **Interviewer Name: _____________________________________** |
| --- |
| **Participant Name: _____________________________________** |
| **Date: _____________________________________** |

**Instructions**

< 5 minutes

***Instructions to the Interviewer:***

*Remember to obtain consent from the interviewee and then start recording the interview.*

***The next 45-60 minutes should include:***

- Introduction and review of “Instructions to the Interviewee” (< 5 minutes)
- Specific Questions and Probing (≈ 40-55 minutes)

***Instructions to the Interviewee:***

- Thank you for taking out time from your schedule for this interview! We will be spending the next 45-60 minutes together. My name is XX and I am a management professor/postdoc/PhD student from University of Colorado Boulder. This study has been approved by the Internal Review Board of CU Boulder and we will follow all guidelines to protect your privacy and confidentiality.
- The purpose of this interview is to explore the downsides of attractiveness in the workplace.
- **Obtaining Verbal Consent**
  - A couple of things before we get started. First, this interview is voluntary. You have the right not to answer any questions, and to stop the interview at any time or for any reason. You will not be compensated for this interview.
  - Second, the questions I will ask are very open-ended. There is no correct answer – I just want to hear what you have to say and learn from you. It’s your opinion that counts.
  - Third, all of your answers are completely confidential (meaning only the research team will see your individual responses).
  - Forth, with your permission, I will be recording your answers during the interview. Again, what you share with me today will be held in confidence, except if you give me permission to share your response with another person. You also have the right to revoke recording permission and/or end the interview at any time.
  - Does all of that sound okay? Do you have any questions now? Do you consent to participate in this study?

≈ 45-55 Minutes

**Questions for Participants**

1. How important has your appearance been to your personal and professional life?
2. Do you think attractiveness affects self-esteem?
3. How do you balance your role as an attractive woman and a professional woman?

4. We are interested in learning whether there are any unseen costs of attractiveness. Can you think of any potential costs of being an attractive woman at work? Do you have any examples pertaining to yourself or others?

5. Have you ever worked with someone who was attractive and you think it had negative repercussions for her?

6. Have you ever used your looks to get ahead?

7. One stereotype is that other women feel a lot of negativity toward attractive women. Can you speak to that at all?

6. Do you think being attractive makes women feel objectified, question their own level of competence, second-guess their abilities or make them self-conscious?

8. *Demographics*

Age, race, level of education, work (full time, part time, etc.), industry, work role
