# Supplementary material for: Women at work: Changes in sexual harassment between September 2016 and September 2018
Source: PLoS One. 2019 Jul 17;14(7):e0218313. doi: 10.1371/journal.pone.0218313 (PMC6636712; doi:10.1371/journal.pone.0218313)
Supplement: S2 File — Interview questions and protocol for 2018 qualitative interviews. (DOCX) [file pone.0218313.s002.docx]

University of Colorado at Boulder

LEEDS School of Business

**Interview Protocol**

| **Interviewer Name: _____________________________________** |
| --- |
| **Participant Name: _____________________________________** |
| **Date: _____________________________________** |

**Instructions**

< 3 minutes

***Instructions to the Interviewer:***

*Remember to obtain consent from the interviewee and then start recording the interview.*

***The next 15-20 minutes should include:***

- Introduction and review of “Instructions to the Interviewee” (< 3 minutes)
- Specific Questions and Probing (≈ 12-17 minutes)

***Instructions to the Interviewee:***

- Thank you for taking out time from your schedule for this interview! We will be spending the next 15-20 minutes together. My name is XX and I am a management professor/postdoc/PhD student from University of Colorado Boulder. This study has been approved by the Internal Review Board of CU Boulder and we will follow all guidelines to protect your privacy and confidentiality.
- The purpose of this interview is to explore sexual harassment after the #MeToo Movement.
- **Obtaining Verbal Consent**
  - A couple of things before we get started. First, this interview is voluntary. You have the right not to answer any questions, and to stop the interview at any time or for any reason. You will not be compensated for this interview.
  - Second, the questions I will ask are very open-ended. There is no correct answer – I just want to hear what you have to say and learn from you. It’s your opinion that counts.
  - Third, all of your answers are completely confidential (meaning only the research team will see your individual responses).
  - Forth, with your permission, I will be recording your answers during the interview. Again, what you share with me today will be held in confidence, except if you give me permission to share your response with another person. You also have the right to revoke recording permission and/or end the interview at any time.
  - Does all of that sound okay? Do you have any questions now? Do you consent to participate in this study?

≈ 12-17 Minutes

**Questions for Participants**

1

1. Do you feel that sexual harassment in the workplace had changed in the last two years? If so, why?

2. Do you feel that women’s reactions to sexual harassment in the workplace have changed in the last two years?

For the interviewer:

Pull quotes from the participant’s interview in September 2016 about her experiences with sexual harassment in the workplace.
